# Life Cycle Emissions and Driving Forces of Air Pollutants and CO_2_ from Refractory Manufacturing Industry in China Based on LMDI Model

**DOI:** 10.3390/toxics13070533

**Published:** 2025-06-26

**Authors:** Yan Wang, Yu Shangguan, Cheng Wang, Xinyue Zhou, Huanjia Liu, Yi Cao, Xiayu Liu, Yan Guo, Guangxuan Yan, Panru Kang, Ke Cheng

**Affiliations:** 1 School of Public Health, Xinxiang Medical University, Xinxiang 453003, China; kristen213@126.com; 2 School of Environment, Key Laboratory of Yellow River and Huai River Water Environment and Pollution Control, Ministry of Education, Xinxiang 453007, China; 3 Henan Key Laboratory for Environmental Pollution Control, Henan Normal University, Xinxiang 453007, China; 4 China National Environmental Monitoring Centre, Beijing 100012, China; 5 Institute of Infectious Disease Prevention and Control, Luoyang Center for Disease Control and Prevention, Luoyang 471022, China; 6 State Key Laboratory of Environmental Criteria and Risk Assessment, Chinese Research Academy of Environmental Sciences, Beijing 100012, China

**Keywords:** refractory, emission inventory, process-based life cycle assessment, LMDI model, scenario analysis

## Abstract

China is the world’s largest supplier of raw materials and is a major consumer of refractories. The environmental damage that results from the use of refractories has drawn increasing attention. Life cycle emissions of air pollutants and CO_2_ associated with the refractory manufacturing industry between 2009 and 2021 were quantified in this study. Particulate matter, SO_2_, and NO_x_ emissions decreased by 7.1% (1515 t), 23.6% (2982 t), and 27.8% (3178 t), respectively, over the aforementioned period despite refractory output volumes being relatively stable. Advancements in manufacturing and purification technologies and internal modifications within the industry played a significant role in these decreases. To sustain output while significantly lowering emissions, the industry shifted toward the production of new minimally polluting refractories and monolithic refractories and away from the production of highly polluting clay bricks. CO_2_ emission was reduced by 1.36 million tons as a result of product modifications. A logarithmic mean Divisia index (LMDI) model was used to quantify the driving forces of five factors (pollution production coefficient, control technology level, economic development level, economic structure, and consumption structure) affecting emissions. Three different emission reduction scenarios were simulated, and potential emission reductions of 23.1–77.7% by 2030 were projected.

## 1. Introduction

Refractories are widely used in metal and petrochemical production processes and other high-temperature processes [1,2,3]. China had a raw materials output of 24.0 million tons in 2021. At this time, China was the world’s largest supplier of raw materials and a major user of refractories. Refractory manufacturing has grown to become a major industry in Shandong, Liaoning, and Henan provinces [4]. Refractory manufacturing contributes to environmental pollution and influences the climate [2,5,6]. Such manufacturing generates air pollutants such as particulate matter (PM), SO_2_, and NO_x_. These pollutants damage the environment and are harmful to human health [7]. As environmental pollution and global warming have become increasingly considerable worldwide concerns, the prioritization of environmental protection, safety, and health has emerged as a global trend. Refractory and raw material manufacturers have similarly begun prioritizing environmental protection, safety, and health [8,9].

Refractory manufacturing involves various air-polluting operations, including material shredding, calcining, packaging, and transportation. Calcining, which involves the combustion of fossil fuels, generates a substantial amount of gaseous pollutants [10]. In 2005, China consumed more than 70 million tons of coal equivalents in its clay brick production according to the General Office of the State Council of the People’s Republic of China. During calcining, high-temperature reactions between refractories and air occur. These reactions generate PM and NO_x_. With the proposal of the goal of “reaching the peak of carbon and carbon neutrality”, various policies such as energy conservation and carbon reduction continue to be deeply implemented, and the structural reform of the upstream and downstream supply side is deeply promoted. The CO_2_ of refractories, a key industry, deserve attention.

Refractory manufacturers have traditionally invested little in environmental protection, and small refractory manufacturers used to directly discharge dust, noise, gas, and wastewater into the environment. In the early 2000s, the Chinese government implemented a series of environmental protection measures, following which all major refractory production provinces—including Beijing, Liaoning, Henan, and Hebei—established air pollution control standards, policies, and regulations for their local refractory industries [11,12]. Local governments then researched and analyzed their respective refractory manufacturing industry, combined national policies with local economic and environmental conditions, and decided on the types of pollutants that should be controlled, methods of pollution treatment, and pollution emission limits for local enterprises. Pollution originating from refractory manufacturing has been reduced by the implementation of *Technical specifications for used refractory material recycling and utilization* (YB/T 4858—2020) [13,14]. According to the white paper on *China’s Policies and Actions to Address Climate Change* (2021), China has significantly reduced its carbon emission intensity while maintaining healthy economic and social development. In 2020, China’s carbon intensity was reduced by 48.4% from that of 2005, exceeding the target of a 40–45% reduction by 2020 that China had committed to the international community, and saving 5.8 billion tons of CO_2_ emissions in total, basically reversing the situation of rapid growth in CO_2_ emissions. [15,16]. Few studies have investigated the Chinese refractory industry’s contribution to air pollution and CO_2_ reduction.

This study adopted the “top-down” life cycle method to calculate the annual air pollutant emissions inventory of refractory material production across the country from 2009 to 2021 and explored the pollutant contributions and changes of different types of refractory materials. In addition, pollution characteristic analyses were conducted on various refractory production processes, including kiln types and fuel types. This is conducive to making an objective assessment of the effect of environmental protection policies in this industry and providing useful information for the future development of this industry and the control of air pollution.

## 2. Data and Methods

### 2.1. Data Sources

Annual output data for refractories and raw materials production were obtained from *China refractory industry yearbooks* for the periods 2010–2014 and 2015–2018 and from the Association of China Refractories Industry. Annual refractory production volumes are illustrated in Figure S1. Data used to compute furnace emissions were obtained from the *Pollutant Generation and Discharge of Industrial Products*. The PM, SO_2_, and NO_x_ removal rates (R) recommended by the “*Jiangsu Province Emission Standard of Air Pollutants for Industrial Furnace* and *Kiln and Technical Guidelines for the Development of Emergency Emission Reduction Measures for Key Industries in Heavy Air Pollution* (2020)” were used. Air pollutant emissions data for 2009–2021 were obtained from the Ministry of Ecology and Environment’s *Report on the State of Ecology and Environment in China*. Energy usage data were obtained from the Refractory Industry Standards Conditions. Calorific value data on energy conversion were obtained from the National Bureau of Statistics’ *Energy Statistics Knowledge Handbook*. Air pollutant emission factors from the *Emission Standard of Air Pollutants for Thermal Power Plants* and related studies on electricity-powered industrial furnaces. CO_2_ emission factor was obtained from the “*Trial Guidelines for Accounting and Reporting Greenhouse Gas Emissions*” of China Power Generation Enterprises.

### 2.2. Methods

#### 2.2.1. Process-Based Life Cycle Assessment

**Figure 1:**
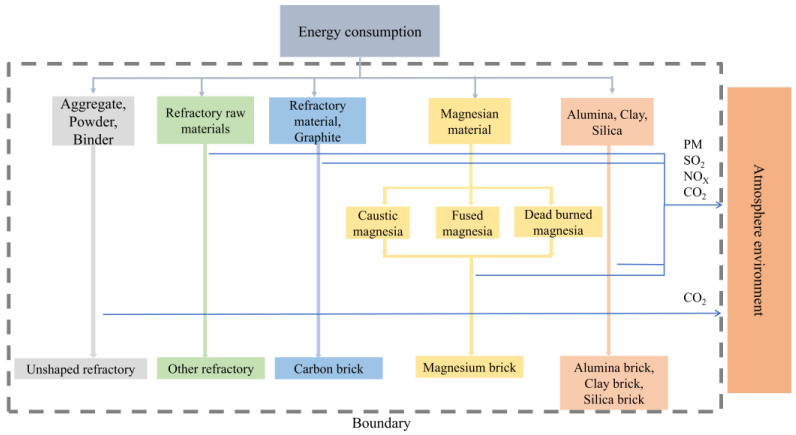
The main production process of refractory industry(Note: Different colors are used to distinguish different stages of the lifecycle).

The term “life cycle” refers to the full primary operations involved in a product’s lifespan, from manufacturing to discontinuation and disposal [17,18]. Life cycle assessment is a standard approach for evaluating the environmental effects of products and systems “from cradle to grave” [19,20]. Process-based life cycle assessment focuses on inventory analysis [21,22]. The life cycle assessment approach has been widely used in China to assess the environmental effects of products and services [23]. A wide variety of refractory goods are available, each with their own set of raw ingredients [24,25,26,27], and this is shown in Figure 1. Furthermore, various types of production furnace exist, each employing their own energy sources. Furnaces can be powered by coal, gas, oil, or electricity. Life cycle assessment aids in depicting the comprehensive pollution scenario in the refractory manufacturing industry. Life cycle assessment comprises three perspectives: the energy source, raw materials, and product. Most life cycle assessment studies have concentrated on a single type of refractory [8] or a single type of pollutant [10,28,29]. Few studies have investigated time and spatial distributions of pollutants in the sector overall.

A process-based life cycle assessment approach was employed in this study to determine emissions inventories for various types of refractory manufacturing for the period 2009–2021. The emissions characteristics of several refractory manufacturing processes, including the effects of furnace type and fuel type on these characteristics, were investigated. Emission reduction scenarios were developed, and air pollution trends were forecast. The effects of environmental protection laws were evaluated, and valuable insights were obtained. The findings of this study may be used to improve air pollution management policies. Due to the excessive complexity of modeling entire life cycle systems and insufficient data regarding product disposal, this study analyzed only the effects of raw material acquisition and refractory production.

#### 2.2.2. System Boundary

This study investigated emissions generated by primary manufacturing processes used in the refractory manufacturing industry; these processes are illustrated in Figure 1.

The system boundary in the figure clearly defines the scope covered by the LCA of the refractory materials production process [30], and its specific connotation is as follows: For magnesia synthetic materials such as re-burned magnesia sand, electrically fused magnesia sand, and light-burned magnesia, the raw material synthesis steps involved in their production process, including high-temperature treatment processes such as ore calcination and electric melting, as well as the chemical reaction synthesis of raw materials, are all included within the system boundary. These processes consume a large amount of energy and produce waste gas emissions [31].

In the production and manufacturing process, various raw materials such as alumina, clay, silica, and magnesia, which have undergone pre-treatment, are fired in high-temperature kilns to obtain the desired physical and chemical properties. The fuel consumption of the kiln (such as natural gas, coal, etc.) and the large amount of waste gas emissions (including pollutants such as PM, SO_2_, NO_x_, CO_2_, etc.) produced during the combustion process are important considerations within the system boundary of this link [8,32]. For some refractory materials with special requirements, subsequent heat treatment processes may also be involved, which are also included within the system boundary. In product output and waste disposal.

During the process, after a series of production procedures, various refractory products such as alumina bricks, clay bricks, silica bricks, magnesia bricks, carbon bricks, and unshaped refractory materials are finally formed and are output as the system within the boundary range [33].

By clearly defining the system boundaries, a comprehensive and accurate life cycle assessment of the refractory materials production process can be conducted, identifying the resource and energy consumption and environmental impact of each link, and providing a decision-making basis for the sustainable development of the industry.

Emissions from the manufacturing of dense and shaped refractories, unshaped refractories, and other refractories were investigated. Indirect emissions stemming from the energy consumption associated with refractory manufacturing were also accounted for.

#### 2.2.3. Calculation Method

Emission inventories for air pollution sources are commonly developed using either a top-down or bottom-up methodology [34,35]. The bottom-up approach involves calculating emissions of various pollutants from individual sources on the basis of survey or monitoring data collected at each source. By contrast, the top-down approach involves calculating emissions from different sources by applying emission factors to activity level data obtained from statistical sources such as yearbooks [36]. China’s refractory manufacturing industry is highly decentralized, with numerous active manufacturers, which makes bottom-up assessment of emission inventory challenging. Consequently, this study employed a top-down approach. Pollution from three sources was analyzed: direct emissions from the manufacture of refractory final products, indirect emissions from the manufacture of refractory raw materials, and indirect emissions from the energy required in the aforementioned manufacturing processes.

Annual emissions of PM, SO_2_, and NO_x_ were quantified. Direct emissions from production processes and indirect emissions from raw material production processes were quantified using Equation (1). The emissions included for analysis comprised both emissions from manufacturing processes and emissions from the energy consumption associated with the manufacturing processes.

(1) $$E_{k}=\sum_{i=1}E_{i,product}+E_{i,material}$$

where $E_{k}$ represents the total amount of air pollutant *k* emitted annually by refractories, $E_{i,product}$ represents the direct emissions of refractory type *i*, and $E_{i,\;material}$ represents the indirect emissions corresponding to the raw materials consumed by refractory product *i* (with all emissions in unit of tons).

$E_{i,\;product}$ was calculated as follows:

(2) $$E_{i,product}=A_{i}\times EF_{i,k}\times (1-R_{i,k})\times 10^{-3}$$

where $A_{i}$ represents the annual output of refractory *i* (unit: t), ${EF}_{i,k}$ represents the emission coefficient of pollutant *k* corresponding to the production of refractory type *i* (kg t^−1^), and $R_{i,k}$ represents the rate of removal (%) of pollutant *k* by flue gas treatment facilities.

For PM, SO_2_, and NO_x_, the amount of pollutant $E_{i,\;material}$ corresponding to the raw materials consumed by refractory *i* was calculated as follows:

(3) $$E_{i,material}=\sum_{j=1}A_{i}\times AF_{i,j}\times EF_{j,k}\times (1-R_{i,j,k})\times 10^{-3}$$

where ${AF}_{i,j}$ represents the conversion coefficient (t t^−1^) of raw material type *j* required to produce 1 ton of type *i* refractory, ${EF}_{i,j,k}$ represents the emission coefficient of pollutant *k* in the production of raw material *j* (kg t^−1^), and $R_{i,j,k}$ represents the rate of removal (%) of pollutant *k* by the flue gas treatment facility in the production of raw material *j*. The production coefficient selected for use in the calculations was that provided in the manual of the Pollutant Generation and Discharge of Industrial Products, and the removal efficiency of each pollutant was determined on the basis of the Jiangsu Province Emission Standard of Air Pollutants for Industrial Furnaces and Kilns. The production coefficient and removal efficiency of pollutants are shown in Tables S1 and S2. Due to a lack of data, average pollution coefficients for each type of refractory material product were calculated. The pollution coefficients in the present study differ from those in other studies because CO_2_ emissions data related to the manufacture of refractories were not available. CO_2_ from refractory manufacturing primarily originate from the burning of fossil fuels. Fossil fuels are burned to provide energy for furnaces. The amount of fuel or electricity consumed by furnaces was determined using the heating coefficient. The analysis accounted for the overall energy consumption required for refractory and raw material production and the unit energy consumption per product. Additionally, CO_2_ emissions associated with energy consumption were taken into account and calculated as follows:

(4) $$C=\sum_{i=1}C_{i,product}+C_{i,matertial}$$

where C is the annual CO_2_ emissions of the refractory manufacturing industry, $C_{i,product}$ represents the CO_2_ emissions generated in the production of refractory products, and $C_{i,\;material}$ represents the CO_2_ emissions generated in the production of refractory raw materials (including dead burnt magnesia, fused magnesia, caustic calcined magnesia). Refractory manufacturers do not currently manage CO_2_ emissions; thus, the calculation of CO_2_ emissions did not consider removal efficiency.

CO_2_ emissions associated with the production of refractory products and raw materials (i.e., $C_{i,\;product}$) were calculated using Equations (5) and (6), respectively.

(5) $$C_{i,product}=\frac{A_{i}\times PC_{i}}{J_{f}}\times EF_{f}\times 10^{-3}$$

(6) $$C_{i,material}=\sum_{j=1}\frac{A_{i}\times AF_{i,j}\times PC_{j}}{J_{f}}\times EF_{f}\times 10^{-3}$$

where ${PC}_{i}$ represents the energy consumption value per unit refractory product (kJ t^−1^), $J_{f}$ represents the unit heating value (kJ) of the energy used by the furnace, and ${EF}_{f}$ represents the CO_2_ emission coefficient of the energy used (kg t^−1^). The CO_2_ calculation parameters are shown in Table S3. Activity level data are shown in Tables S4 and S5. The level of control technology used in each process and the condition of equipment varied, and relevant data of the application ratio of control technology were lacking. In 2020, the PM, SO_2_, and NO_x_ removal efficiencies related to the refractory manufacturing industry in Henan Province were 65%, 85%, and 75%, respectively.

### 2.3. Driving Factor Analysis

The logarithmic mean Divisia index factor decomposition method was employed to quantitatively analyze the factors influencing air pollutant emissions in the refractory manufacturing industry [37]. The logarithmic mean Divisia index (LMDI) is based on the Kaya identity [38]. In the present study, the pollution production coefficient *f*, control technology level *q*, economic development level (i.e., *GDP*), economic structure *e*, and refractory consumption structure *s* were employed to quantitatively analyze the factors influencing air pollutant emissions in the refractory manufacturing industry. In this study, the Kaya identity is generalized and an equation suitable for this study is obtained:

(7) $$E=f\times \left(1-\eta \right)\times GDP\times \frac{A_{steel}}{GDP}\times \frac{A_{i}}{A_{steel}}=f\times q\times GDP\times e\times s\;$$

where *η* represents the comprehensive removal efficiency of control technology, *q* = 1 − *η* represents the upgrading of control technology, *e* = *A_steel_*/*GDP* represents the ratio of crude steel production to *GDP* due to policy changes in China’s steel industry, and *s* = *A_i_*/*A_steel_* represents the effect of the main downstream industry (steel industry) on the output of the refractory manufacturing industry. This led to

(8) $$lnE=B+\alpha_{1}lnf+\alpha_{2}lnq{+\alpha }_{3}lnGDP+\alpha_{4}lne{+\alpha }_{5}lns$$

From formula (8), the following can be obtained:

(9) $$\begin{array}{ll} \Delta E=E^{T}-E^{0}= & f^{T}q^{T}{GDP}^{T}e^{T}s^{T}-{f^{0}q}^{0}{GDP}^{0}e^{0}s^{0}\\ & =f_{effect}+q_{effect}+{GDP}_{effect}+e_{effect}{+s}_{effect}\\ & =\alpha_{i}\left(T\right)\ln\left(\frac{f^{T}}{f^{0}}\right)+\alpha_{i}\left(T\right)\ln\left(\frac{q^{T}}{q^{0}}\right)+\alpha_{i}\left(T\right)\ln\left(\frac{{GDP}^{T}}{{GDP}^{0}}\right)\\ & +\alpha_{i}\left(T\right)\ln\left(\frac{e^{T}}{e^{0}}\right)+\alpha_{i}\left(T\right)\ln\left(\frac{s^{T}}{s^{0}}\right) \end{array}$$

(10) $$\alpha_{i}\left(T\right)=\frac{E^{T}-E^{0}}{lnE^{T}-lnE^{0}}.$$

In the LMDI factor decomposition model, the decomposition period is assumed to be [0.T]. *E^T^* represents the pollutant emission of resistant materials in period *T*, and *E*^0^ represents the pollutant emission of resistant materials in the initial period. Δ*E* represents the change in total emissions in the refractory manufacturing industry. This value is equal to the sum of the effects of various influencing factors. Furthermore, *f_effect_*, *q_effect_*, *GDP_effect_*, *s_effect_*, and *e_effect_* represent the changes in the pollutant discharge caused by the pollution production coefficient, control technology level, economic development level, economic structure, and downstream demand change, respectively. These values were estimated using Equations (11)–(15).

(11) $$f_{effect}=\frac{E^{T}-E^{0}}{\ln E^{T}-\ln E^{0}}\times \ln\frac{f^{T}}{f^{0}}$$

(12) $$q_{effect}=\frac{E^{T}-E^{0}}{\ln E^{T}-\ln E^{0}}\times \ln\frac{q^{T}}{q^{0}}$$

(13) $${GDP}_{effect}=\frac{E^{T}-E^{0}}{\ln E^{T}-\ln E^{0}}\times \ln\frac{{GDP}^{T}}{{GDP}^{0}}$$

(14) $$e_{effect}=\frac{E^{T}-E^{0}}{\ln E^{T}-\ln E^{0}}\times \ln\frac{e^{T}}{e^{0}}$$

(15) $$s_{effect}=\frac{E^{T}-E^{0}}{\ln E^{T}-\ln E^{0}}\times \ln\frac{s^{T}}{s^{0}}$$

The contribution of various factors to the change in pollutant emissions was subsequently determined. A positive value meant that the pollutant emissions had increased, and a negative value meant that the emissions had decreased.

### 2.4. Emission Reduction Scenario

This study examined existing emission control methods, changes in industrial output, and potential future directions for low-carbon policies and technological advancements. To estimate air pollutant emissions and identify the most appropriate CO_2_ reduction methods in the refractory manufacturing industry in 2025 and 2030, a baseline scenario and two emission reduction scenarios were defined using 2020 as the base year [39,40]. The baseline scenario assumed that the control technology employed by the refractory manufacturing industry would remain at the 2020 level. The output of different product categories in the base year was projected to increase or decrease, and the potential for emission reduction through changes in the industry’s structure was examined. In the first emission reduction scenario, new laws and regulations are passed and emission limits are progressively tightened, resulting in the broad implementation of control facilities and the modernization of treatment technologies. In the second emission reduction scenario, even stricter laws and regulations and more advanced control technology are implemented than the first emission reduction scenario. The first emission reduction scenario is the most likely future scenario because it accounts for recent emissions criteria for refractory manufacturing and related policy planning [40]. The second emission reduction scenario involves stricter regulations regarding production processes and anticipates the implementation of more efficient treatment methods compared with the first emission reduction scenario. The predicted rate of removal of each pollutant is shown in Table S6, and the output for each type of refractory is shown in Table S7.

## 3. Results and Discussion

### 3.1. Pollution Characteristics of Refractory Furnaces

**Figure 2:**
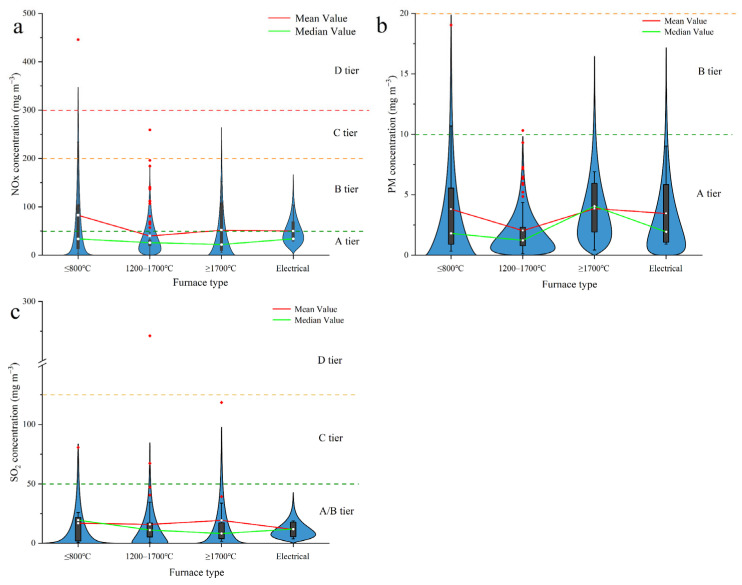
Distribution of average emission concentrations of air pollutants by different furnace type: (**a**) PM, (**b**) SO_2_, and (**c**) NO_x_.

PM, SO_2_, and NO_x_ emissions were analyzed using automatic monitoring station data obtained from 150 refractory production companies in Henan, China. In the *Technical Guidelines for the Development of Emergency Emission Reduction Measures for Key Industries in Heavy Air Pollution* (2020), the existing enterprises are divided into four tiers of A, B, C, and D for the performance classification index of the refractory industry. The emission limits for each class are shown in the figure. The average concentration of PM in emissions from these companies in 2020 was 2.59 mg m^−3^ (Figure 2a). The average concentrations of PM in emissions from the fuel burning furnaces operated at temperatures of ≤800, 1200–1700, and ≥1700 °C were 3.81, 2.03, and 3.85 mg m^−3^, respectively. Conversely, the average concentration of PM in emissions from electric furnaces was 3.46 mg m^−3^. The emission limit in Henan Province is 10 mg m^−3^ (Emission Standard of Air Pollutants for Industrial Kiln and Furnace; DB41 1066–2020 [41]).

In Figure 2a, the lowest and highest average PM concentrations among the 21 furnaces with a temperature of ≤800 °C were 0.35 and 19.04 mg m^−3^, respectively, and the median concentration was 1.81 mg m^−3^. Of the four types of furnaces (those operated in the aforementioned three ranges and those powered electrically), those operating at the lowest temperatures had the widest distribution of PM concentrations. In total, 86 companies used furnaces that operated at 1200–1700 °C; the lowest and highest PM concentrations for these furnaces were 0.14 and 10.32 mg m^−3^, respectively, and the median concentration was 1.24 mg m^−3^. Nonetheless, of the four types of furnaces, those that operated at 1200–1700 °C emitted the smallest amounts of overall pollutants and had the most concentrated distribution (primarily in the range 0–5.0 mg m^−3^).

The lowest and highest average PM concentrations for the 15 furnaces operated at a temperature of ≥1700 °C were 0.43 and 6.92 mg m^−3^, respectively, and the median concentration was 4.06 mg m^−3^. The median PM concentration for the four electric furnaces was 1.94 mg m^−3^, and the lowest and highest average concentrations were 0.92 and 9.04 mg m^−3^, respectively. Electric furnaces do not burn fossil fuels; however, the amount of PM that the electric furnaces emitted was generally the same as that emitted by the fuel furnaces. This indicates that fuel combustion is not the sole source of the PM in emissions from refractory manufacturers. PM released from raw materials at high temperature contributes considerably to overall PM emissions.

Bag filters are currently the most frequently used PM processing technology in the refractory manufacturing industry. These filters have dust removal efficiency of 99.9%. According to the findings of the present study, refractory manufacturers can comply with the PM concentration limit of <10 mg m^−3^ by using bag filters.

In Figure 2b, the SO_2_ emission concentrations from January to October 2020 are shown in Figure 2b. For furnaces operated at temperatures of ≤800, 1200–1700, and ≥1700 °C, the average concentrations of SO_2_ were 16.9, 15.9, and 19.3 mg m^−3^, respectively. The average concentration of SO_2_ in electric furnaces was 6.16 mg m^−3^.

For the furnaces operated at ≤800 °C, the lowest and highest average SO_2_ concentrations were 0.51 and 80.79 mg m^−3^, respectively, and the median was 19.5 mg m^−3^. In total, 84 companies employed furnaces that operated at 1200–1700 °C; the median SO_2_ concentration was 11.2 mg m^−3^, and the lowest and highest concentrations were 0.30 and 271 mg m^−3^, respectively. The furnaces operated at 1200–1700 °C were the most numerous; their average SO_2_ concentration was the lowest of the four types of furnaces, and their SO_2_ concentration distribution was relatively narrow.

In total, 15 furnaces operated at temperatures of ≥1700 °C. The lowest and highest average SO_2_ emission concentrations for these furnaces were 1.72 and 118.5 mg m^−3^, respectively, and the median emission concentration was 8.28 mg m^−3^. Four electric furnaces operated at high temperature were also examined. Of the four furnace types, electric furnaces emitted the least SO_2_, probably because electric furnaces do not burn fossil fuels.

In Figure 2c, NO_x_ emission concentrations in 2020 are shown in Figure 2c. Fuel furnaces with temperatures of ≤800, 1200–1700, and ≥1700 °C had average NO_x_ concentrations of 82.5, 39.8, and 51.7 mg m^−3^, respectively, and electric furnaces had an average NO_x_ concentration of 50.2 mg m^−3^. In total, 15 furnaces operated at temperatures of ≤800 °C. The median average concentration was 33.8 mg m^−3^, and the lowest and highest concentrations were 3.70 and 445 mg m^−3^, respectively. In total, 86 furnaces operated at temperatures of 1200–1700 °C. The median NO_x_ concentration was 26.0 mg m^−3^, and the lowest and highest values were 0.78 and 259 mg m^−3^, respectively. Due to the widespread use of this type of furnace and the NO_x_ values being concentrated in the low range, the average NO_x_ concentration was lowest for this type of furnace.

In total, 15 furnaces operated at temperatures of ≥1700 °C. The median NO_x_ emission concentration was 8.28 mg m^−3^, and the lowest and highest values were 6.36 and 152 mg m^−3^, respectively. The lowest and highest average NO_x_ emission concentrations for the four high-temperature electric furnaces were 29.0 and 103.4 mg m^−3^, respectively, and the median concentration was 22.2 mg m^−3^. The NO_x_ emissions from the electric furnaces were the same as those from the fuel furnaces despite the fact that electric furnaces do not burn fossil fuels.

Furnaces operated at 1200–1700 °C were found to be the least polluting of the furnaces that analyzed. This is most likely because these furnaces, which are widely used, have advanced pollution control systems and stringent oversight protocols. Nonetheless, several of the furnaces analyzed that operated at 1200–1700 °C were discovered to emit large amounts of pollutants. Few of the furnaces investigated in this study operated at ≤800 or ≥1700 °C. The distribution of emission concentrations for these furnace types was relatively wide, and these furnace types had higher average emission concentrations for all three pollutants studied. Furnaces operated at ≤800 °C use less fuel but have higher average emission concentrations. Of the four types of furnaces, the high-temperature electric furnaces had the lowest emission concentrations (except for PM, which was emitted to the same degree by electric furnaces as by the other types of furnaces) because they did not burn fossil fuels.

### 3.2. Temporal Emissions from Refractory Industry

During the last years of the 11th Five-Year Plan (2006–2010), refractory production in China increased. During the 12th Five-Year Plan (2011–2015), refractory production started decreasing. China’s refractory production reached a peak of 29.5 million tons in 2011 and had dropped to 26.2 million tons by 2015, representing a decrease of 11.3%. During the 13th Five-Year Plan (2015–2020), China’s refractory production averaged 23.9 million tons per year. One of the main causes of the discovered variations in pollutant emissions was the variations in annual refractory production.

**Figure 3:**
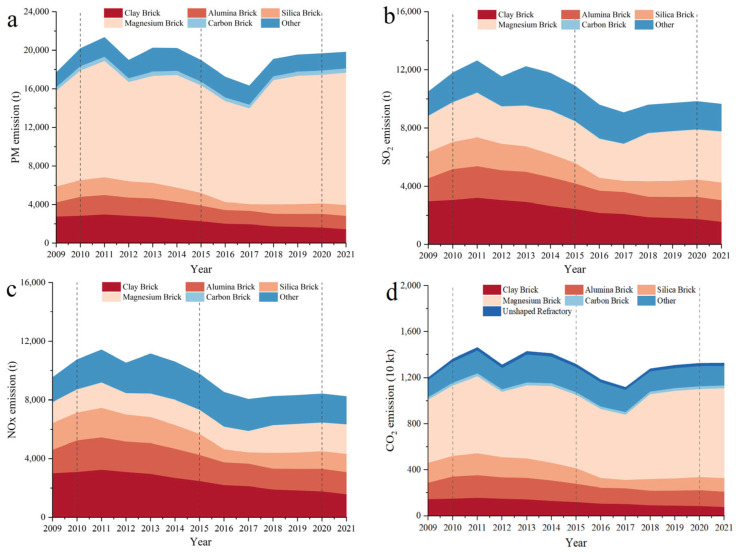
Air pollutants and CO_2_ of refractory products, 2009–2021: (**a**) PM, (**b**) SO_2_, (**c**) NO_x_, and (**d**) CO_2_.

PM emissions from refractory manufacturing peaked in 2011 at approximately 21,334 t year^−1^, which represents 0.19% of China’s total PM emissions (Figure 3a). The total PM emissions of the refractory manufacturing industry sharply declined at the start of the 12th Five-Year Plan, reaching approximately 18,958 t year^−1^ in 2015 (0.15% of all PM emissions). PM emissions from the refractory manufacturing industry in 2015 were 13.1% lower than in 2011. During the 13th Five-Year Plan (2016–2020), overall PM emissions decreased significantly before plateauing. In 2020, PM emissions were 19,660 t year^−1^. PM emissions increased somewhat at the start of the 14th Five-Year Plan, reaching 19,816 t in total in 2021. During the 11th Five-Year Plan (2006–2010), advanced PM treatment technologies were developed. Variation in annual production is a key factor affecting emissions.

PM emissions in China have been steadily declining as the use of extremely polluting clay bricks has been increasingly outlawed by national legislation. The PM emissions associated with clay bricks accounted for 13.8% of the total PM emissions caused by refractory production 13.8% in 2011, 11.8% in 2015, 8.1% in 2020, and 7.2% in 2021. The emissions associated with high-alumina bricks, which are of higher quality than clay bricks, remained mostly constant during the 12th and 13th Five-Year Plans (9.4% in 2011 and 7.2% in 2020). The emissions associated with silica bricks were 9.4% in 2011, 8.6% in 2015, and 6.9% in 2021. The production of raw materials generates more pollutants, which is why the emissions associated with magnesium bricks increased from the 11th to the 14th Five-Year Plans, being 56.2% in 2009 and 69.2% in 2021. During the 12th Five-Year Plan, the PM emissions associated with carbon bricks were relatively low and constant, being 2.09% in 2015, 2.24% in 2020, and 2.19% in 2021. The emissions associated with other refractories varied during the 13th Five-Year Plan, increasing from 9.5% to 11.8% during the 12th Five-Year Plan and being 12.5% in 2016 and 8.8% in 2021.

China achieved effective control over PM emissions from its refractory manufacturing during the 12th and 13th Five-Year Plans, resulting in emission reductions of 11.1% and 19.7%, respectively. The market for the three primary traditional refractories—clay, high-alumina, and silica bricks—became saturated resulting in the effective control of PM emissions. At the same time, refractory manufacturing enterprises are starting to develop new refractories. Fume control for specific new refractories should also be paid attention to.

SO_2_ emissions between 2009 and 2021, illustrated in Figure 3b, appear to have no correlation with production. In 2011, SO_2_ emissions from the refractory manufacturing industry reached 12,624 t year^−1^. Due to a decrease in refractory production, SO_2_ emissions started to decrease during the 12th Five-Year Plan. At the end of the 12th Five-Year Plan, SO_2_ emissions were 10,898 t year^−1^, which represents a decrease of 13.7%. During the 13th Five-Year Plan, the government clamped down on SO_2_ emissions. In 2017, SO_2_ emissions were 9054 t year^−1^, which was a 16.9% decrease from 2015. In 2021, SO_2_ emissions were 9642 t year^−1^.

During the 2009–2015, processes related to the production of clay, magnesium, silica, and high-alumina bricks were the primary sources of SO_2_ emissions. In 2011, the SO_2_ emissions associated with the production of these four types of bricks were 25.2%, 24.2%, 17.2%, and 15.7%, respectively, accounting for 82.3% of all SO_2_ emissions. Clay, high-alumina, and silica bricks all had higher SO_2_ emission rates than PM emission rates because these bricks were produced using coal as the primary energy source and coal has a higher SO_2_ emission factor than gas and oil. During the 12th Five-Year Plan, clay bricks were progressively phased out, and emissions associated with clay bricks started to decline. Emissions associated with clay bricks were 25.2% in 2011 and 22.2% in 2015. During the 13th Five-Year Plan, the emissions associated with clay bricks continued to decline, reaching 17.5% in 2020. During the 14th Five-Year Plan, the emissions associated with clay bricks continued to decline, reaching 15.9% in 2021. During the 12th and 13th Five-Year Plans, the emissions from high-alumina bricks were constant. The SO_2_ emissions associated with silica bricks were 15.7% in 2011, 8.4% in 2017, and 12.7% in 2021. Production of magnesium bricks has recently increased, resulting in an increase in SO_2_ emissions. The SO_2_ emissions associated with magnesium bricks were 23.7% in 2009 and 26.2% in 2015. The magnesium brick industry grew quickly during the 13th Five-Year Plan, and in 2021, the SO_2_ emissions associated with magnesium bricks were 36.4%. The emissions associated with these four refractories were 80.2% in 2021, down 2.2% from 2011. Since no fossil fuels were burned during the manufacturing of carbon bricks, the SO_2_ emissions associated with carbon bricks were minimal (less than 1.0% on average). Magnesium brick output has increased, and consequently, SO_2_ emissions have increased. In 2009 and 2015, sulfur dioxide emissions accounted for 15.8% and 22.2% of the total SO_2_ emissions of resistant materials, respectively. During the “13th Five-Year Plan” period, the output of various refractories was stable. It peaked at 24.2% in 2016 and then began to decline to 19.5% in 2021. This demonstrates that refractory manufacturers are producing increasingly more types of refractories, in line with laws protecting the environment and consumer demand. Production of other refractories and their associated SO_2_ emissions have started to increase; however, the production market for clay brick, high-alumina brick, and silica brick refractories has started to reach a stable state. In these cases, the corresponding emission standards should be established on the basis of emissions profiles for single refractory types.

NO_x_ emissions are shown in Figure 3c. The trend in NO_x_ emissions is similar to that for SO_2_ emissions. During the “12th Five-Year Plan” period, the emission trend of NOx and SO_2_ emissions are roughly the same, reversing the upward trend and getting a substantial reduction. NO_x_ emissions fell 14.4% from 11,412 t in 2011 to 9770 t in 2015. NO_x_ emissions were 8234 t in 2021.

NO_x_ emissions of the three traditional resistant materials, clay brick, high-aluminum brick and silicon brick, accounted for 67.2% of the total NO_X_ emissions of refractory materials in 2009 and 52.2% in 2021. Magnesium bricks accounted for a progressively larger percentage of NO_x_ emissions—15.0% in 2009 compared with 24.4% in 2021. The proportion of emissions associated with carbon bricks increased from 0.1% in 2009 to 0.2% in 2021. The proportion of NO_x_ emissions associated with magnesium bricks increased from 15.0% in 2009 to 16.7% in 2015 and quickly increased during the 13th Five-Year Plan. The NO_x_ emissions associated with magnesium bricks were responsible for 23.2% of all NO_x_ emissions in 2020 and were responsible for 24.4% of all NO_x_ emissions in 2021. During the 12th Five-Year Plan, other refractory was developed quickly, and the proportion of NO_x_ emissions associated with these other refractory goods increased from 19.5% in 2011 to 25.1% in 2015. During the 13th Five-Year Plan, the proportion of NO_x_ emissions associated with specific refractories varied depending on market demand, reaching a maximum of 27.6% in 2016 and 23.1% in 2021.

CO_2_ emissions are illustrated in Figure 3d. During the 12th Five-Year Plan, output at refractory manufacturers declined, affecting overall CO_2_ emissions. CO_2_ emissions were 14.65 × 10^6^ t year^−1^ in 2011, 13.19 × 10^6^ t year^−1^ in 2015, 11.18 × 10^6^ t year^−1^ in 2017, and 13.29 × 10^6^ t year^−1^ in 2021. The CO_2_ emissions associated with clay, high-alumina, and silica bricks, which are primarily produced using coal and gas, decreased from 37.1% in 2011 and to 25.3% in 2020. In 2020, the CO_2_ emissions associated with clay, high-alumina, and silica bricks were 6.3%, 10.4%, and 8.6%, respectively. The CO_2_ emissions associated with magnesium bricks, which are primarily produced using oil, were 45.7% in 2011 and 58.7% in 2021. The CO_2_ emissions associated with other refractories were 13.6% in 2011 and 13.2% in 2020. Carbon bricks and monolithic refractories were produced in large quantities; however, the production of carbon bricks and monolithic refractories did not substantially contribute to the total CO_2_ emissions because this production does not involve the burning of fossil fuels.

The number of refractories produced in 2009 was comparable to that in 2021; however, emissions substantially decreased in this period. This suggests that the industry matured, producing a more diverse range of products, developing cleaner production methods, and producing products of greater quality as a result of environmental protection legislation. Emissions decreased during the 12th Five-Year Plan. PM, SO_2_, and NO_x_ emissions decreased by more than 10%. Air pollutants discharge was mostly determined by the output of different types of products distribution during the 13th Five-Year Plan, as the refractory industry increasingly consolidated its pollution control measures. In addition, due to the lack of end-treatment measures for CO_2_, the emissions are consistent with the amount of CO_2_ produced during the production of refractory materials. However, the industry’s CO_2_ emissions significantly decreased because of the steady development of cleaner goods, such as monolithic refractories.

### 3.3. Spatial Distribution Characteristics

**Figure 4:**
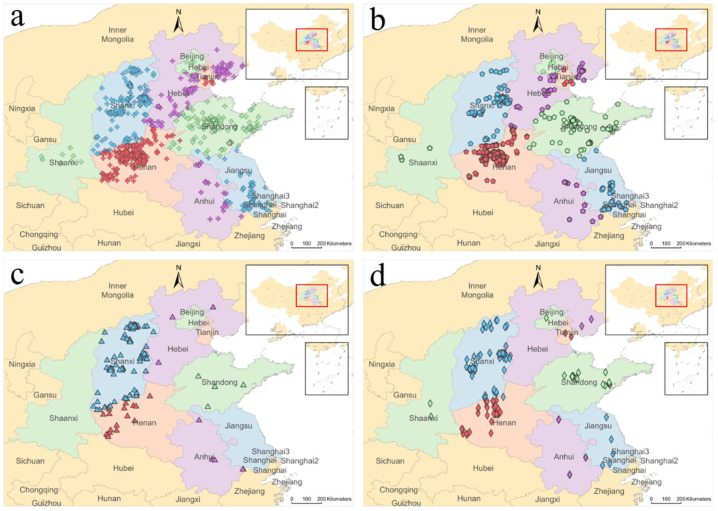
Energy consumption in key areas of refractory production by province: (**a**) electricity, (**b**) gas, (**c**) coal, and (**d**) oil. The symbols in the figure represent the locations of refractory material production enterprises.

The energy consumption of refractory firms in each province was examined, and the usage of electricity, gas, coal, and oil were assessed (Figure 4). Several refractory enterprises are located in North and Northeast China. Enterprises in Henan, Shanxi, Shandong, and Hebei consume more electricity and gas than do enterprises in other provinces. Coal-fired refractory enterprises are concentrated in Shanxi. Oil-fired refractory enterprises are concentrated in Shanxi and Henan.

Most refractory businesses rely on gas and electricity. Consumption of gas and electricity is regulated by the government. The abundance of coal and oil resources in Shanxi Province may be a contributing factor to the use of these resources in that province.

Chinese refractory companies are regionalized. The location of a refractory company is based on resources, markets, technologies, and talent. The geographic dispersion of enterprises is determined by the location of resources. China has a very concentrated magnesite distribution. In 2009, 13% and 86% of the nation’s magnesite was produced in Shandong and Liaoning, respectively. The distribution of bauxite in China is also concentrated. In 2009, 27%, 26%, and 24% of China’s bauxite was produced in Guangxi, Henan, and Guizhou, respectively. Large concentrations of raw material supplies correlated with large concentrations of refractory companies.

**Figure 5:**
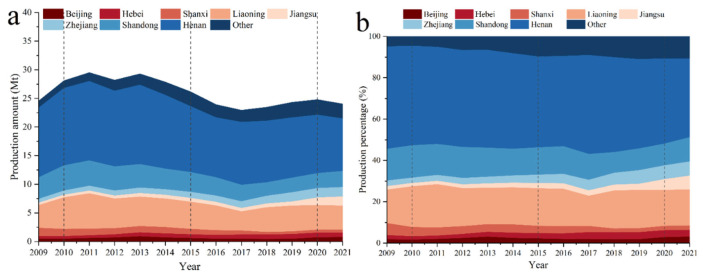
Changes in refractory production in provinces in China, 2009–2021: (**a**) the output of refractory materials in each province, and (**b**) the proportion of refractory materials output in each province.

In Figure 5a, more than 90% of China’s refractory output is from Henan, Liaoning, Shandong, Shanxi, Hebei, Jiangsu, Zhejiang, Shanghai, and Beijing (Figure 5). Most refractories are produced in Henan, Liaoning, and Shandong. In 2010, 48.1%, 19.7%, and 15.7% of China’s total refractory output came from Henan, Liaoning, and Shandong, respectively. In Figure 5b, in 2021, 66.6% of China’s total refractory output came from these three provinces. At the start of the 11th Five-Year Plan, 33.9% (4700 kt) of China’s total refractory output came from Henan, 36.3% (1605 kt) came from Shandong, and 32.1% (1983 kt) came from Liaoning. This may have been due to the more stringent production limitations and business closures in certain provinces throughout the phase of air pollution management. Other provinces absorbed the output and market share that these three provinces left behind. Province-specific variations in raw materials and resources have aided the diversification of refractory products. Liaoning enacted the first local standard in this industry (the Emission Standard of Air Pollutants for Magnesia Refractory Industry). This standard was enacted in 2018, and Liaoning’s output started to rebound, in contrast to that in Henan and Shandong provinces, where production has been continually decreasing. Liaoning’s refractory production increased by 851 kt between 2017 and 2021. This shows that businesses benefit from emissions regulations. Henan Province implemented the Emission Standards of Air Pollutants for Refractory Industry on 1 January 2022. These regulations are expected to improve development in the industry and result in financial gains for businesses in Henan Province.

### 3.4. Uncertainty Analysis

This study quantitatively described the uncertainty in emission inventories by using a Monte Carlo model [42], which can quantify the uncertainty range and transfer uncertainty between variables. Calculations were performed in Oracle Crystal Ball software 11.1.2.4.400 (Oracle, Redwood City, CA, USA) [43]. First, data tables were updated with activity levels (output of different refractory products) and emission factor data. Second, the number of random samples was set to 10,000, with a 95% confidence level. Emission factors and activity intensity were assumed to follow normal distributions. The parameters for each factor applied in this study are shown in Table S8 and are based on earlier research [44]. The defining hypothesis served as the foundation for the probability distribution model, which was then used to calculate the emission inventory, quantitatively transfer uncertainty, and yield the uncertainty interval. The simulated results are displayed in Figure S2.

Two key factors illustrate the uncertainty in the emission inventories. First is the uncertainty in activity levels. Activity level data were obtained from reliable sources (the Association of China Refractories Industry and China Refractory Industry Yearbooks 2010–2014 and 2015–2018); however, human error cannot be ruled out. Second is the uncertainty in emission factors. Emission factors were selected in accordance with the Ministry of Ecology and Environment’s technical guidelines and published publications; however, further research is required to determine the appropriateness of these variables for the refractory industry. This work derived the range of uncertainty in several pollutants (Table 1) by quantitatively analyzing the uncertainty in the emission inventory [45] through a Monte Carlo model. NO_x_ uncertainty was lowest, with an uncertainty interval of −36.9% to 40.1%. PM uncertainty had an uncertainty interval of −49.5% to 54.7%.

### 3.5. LMDI Model-Based Driving Force Analysis

**Figure 6:**
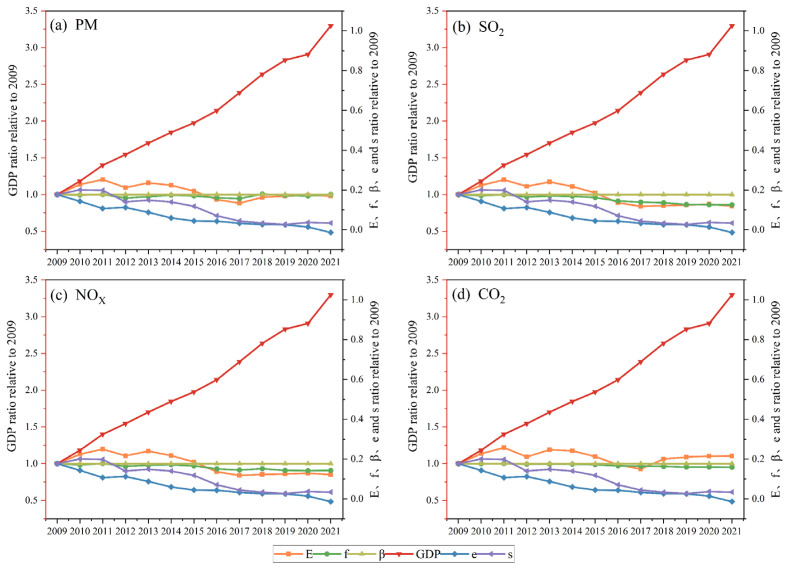
The variation trend of various factor value over the years: (**a**) PM, (**b**) SO_2_, (**c**) NO_x_, and (**d**) CO_2_.

Parameters such as total pollutant emission, control technology level, economic development level (GDP), economic structure, and consumption structure are illustrated in Figure 6. These parameters were normalized such that their value in 2009 was 1. Overall, emissions trended down. From 2009 to 2021, refractory production in China decreased; however, refractory material output did not change. After 2012, under the control of the government, the pollution production coefficient decreased, and source control measures were improved. The emission coefficients of PM, SO_2_, NO_x_, and CO_2_ decreased by 2.1%, 13.9%, 9.7%, and 5.1%, respectively.

**Figure 7:**
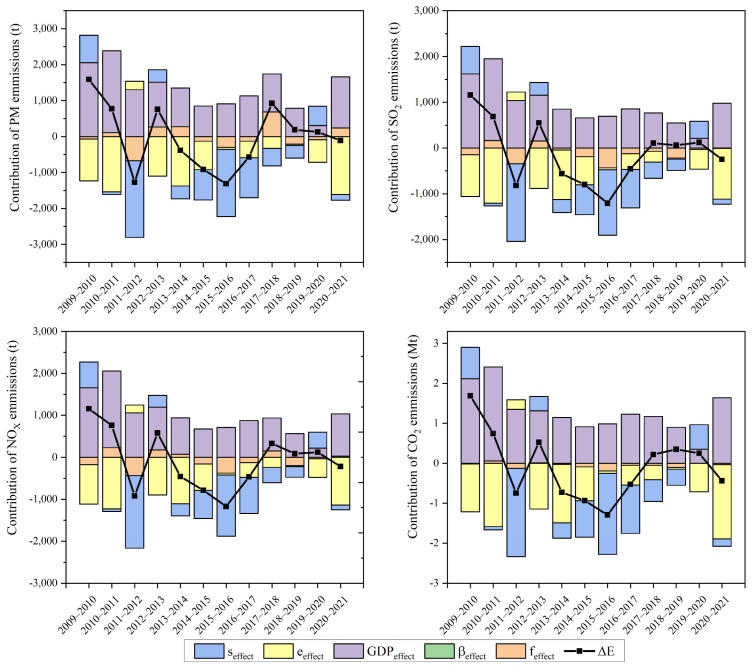
Contribution of each driving factor to air pollutants and CO_2_ emission: PM, SO_2_, NO_x_, and CO_2_.

The contributions of different driving factors to atmospheric pollutants and greenhouse gas emissions are shown in Figure 7. Overall, during the 11th Five-Year Plan, economic development was the dominant factor, leading to an increase in emissions. After 2011, technological innovations and changes in demand offset these increases, leading to a decline in emissions. Economic development was the main factor driving increases in emissions. The gross domestic product (GDP) increased from CNY 3485.2 billion in 2009 to 1,149,237 billion yuan in 2021, an increase of 229.7%. PM, SO_2_, NO_x,_ and CO_2_ emissions increased by 98.1%, 91.1%, 82.6%, and 102.9%, respectively.

The structure of the economy was the main factor that offset increases in PM, SO_2_, NO_x_, and CO_2_ emissions, causing them to decrease. PM, SO_2_, NO_x_, and CO_2_ emissions decreased individually by 65.2%, 60.4%, 59.9%, and 62.5%, respectively.

PM, SO_2_, NO_x_, and CO_2_ emissions were found to be more dependent on economic structure than on consumption structure. Changes in the economic structure have already led to reductions in PM, SO_2_, NO_x,_ and CO_2_ emissions by 45.1%, 42.4%, 39.7%, and 42.2%, respectively, in 2021 compared to 2009.

The changes in influencing factors in 2011, 2012, 2017, and 2018 can be explained by developments in the refractory manufacturing industry in 2011 and 2017. Between 2009 and 2014, the refractory manufacturing industry underwent normative upgrades, and in 2011, refractory production peaked. Localization and production methods, equipment quality, and management styles have all improved over time. Furthermore, the number of enterprises engaged in refractory manufacturing increased in this period. The peak in refractory production in 2011 coincided with a peak in emissions.

In 2017, production restrictions were implemented in Henan, Shandong, and Ningxia, leading to reduced output volumes in that year. By 2018, the market had improved, and increased production was offsetting the effects of a reduced pollution coefficient, resulting in an increase in emissions.

### 3.6. Emission Reduction Projections

The primary factors influencing the amounts of air pollutants released are the economic development level, end control technology level, product structure, and refractory output [46]. The Ministry of Industry and Information Technology seeks to transition the refractory manufacturing industry to a more sustainable model [47]. The refractory manufacturing industry must be tightly controlled, including in the amounts of raw materials it uses. Energy should be conserved, emissions should be reduced, mergers, and reorganization should be avoided, industrial structures should be optimized, the environment should be preserved, and quality and efficiency should be prioritized.

The baseline scenario assumes that control technologies are maintained at the 2020 level and that emission factors and overall output are stable. The output of different goods may increase or decrease, and the structure of refractory products is related to emissions. PM, SO_2_, NO_x_, and CO_2_ emissions are projected to decrease by 1.6%, 16.2%, 21.7%, and 4.3%, respectively, by 2030. Energy consumption limits per unit product for refractory raw materials and for refractory products are specified in the “Standard Conditions for Refractory Industry” (2014) guidelines [48]. Shandong, Liaoning, Henan, and other important provinces that produce refractories have established emissions limits [49,50,51]. PM, SO_2_, and NO_x_ emissions from furnaces belonging to refractory enterprises may not exceed the limits specified in the Technical Guide for the Designation of Emergency Emission Reduction Measures for Key Industries in Heavy Pollution Weather (2020 Revision) [52]. PM, SO_2_, and NO_x_ emissions from Grade A enterprises may not exceed 10, 50, and 50 mg m^−3^, respectively.

The first emission reduction scenario assumes that new laws, regulations, and emissions limits will be introduced. These regulations will result in the replacement of facilities and the modernization of manufacturing processes. By 2025, the refractory industry is expected to primarily be using bag and wet electric dust removal technologies for emissions control. Every business is expected to perform desulfurization and denitrification. Wet desulfurization is the primary method used in desulfurization technology, whereas selective catalytic reduction (SCR), selective non-catalytic reduction (SNCR), and SNCR + SCR are the primary methods used in denitrification technology. Emissions control measures in 2030 will be superior to emissions control measures in 2025. Wet dust collector removal will be phased out, and bag filters and wet electric precipitators will be the primary dust removal facilities. All enterprises will perform wet desulfurization. Denitrification methods will involve SCR; however, several will involve SNCR. The installation of low nitrogen burners will become more common. The governments of Europe, Japan, the United States, and several other countries have prohibited the use of chromium-containing refractories; therefore, development of chromium-free refractory materials is expected to increase. The refractory industry is expected to be chromate-free by 2030. Between 2020 and 2030 for this scenario, PM, SO_2_, NO_x_, and CO_2_ emissions are expected to decrease by 70.9%, 69.4%, 23.1%, and 5.5%, respectively.

**Figure 8:**
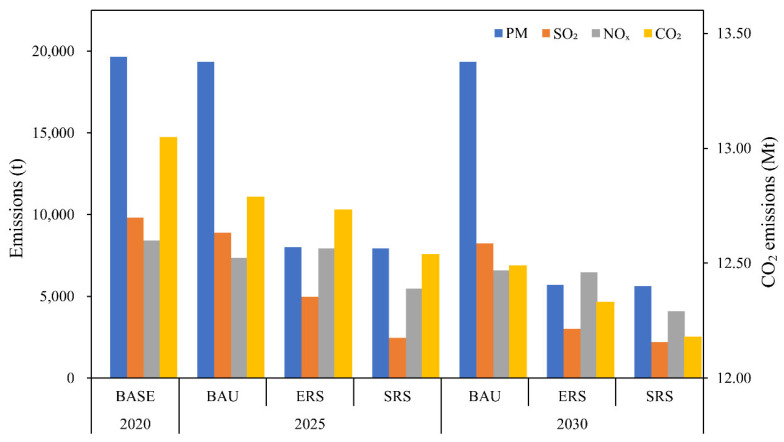
Forecast of air pollutants and CO_2_ emissions in 2025 and 2030 under three emission reduction scenarios.

The second emission reduction scenario assumes the implementation of even stricter regulations and additional improvements in control technologies and governance efficiency. By 2025, control technology will be on par with 2020 levels in strategic regions such as Henan Province, and by 2030, all provinces will be using the optimal control technologies. Stricter emissions limits will be in place. The energy sector has generated nearly 90% of China’s carbon emissions, and the amount of energy emission reduction is huge, and there is still a need to further increase the total energy consumption. The low-carbon development of the future energy field will play a significant role in achieving the goal of “carbon neutrality”. Coal consumption is expected to decrease, thereby reducing carbon emissions. Clean energy consumption will make up a greater proportion of total energy consumption. Use of natural gas will be 10% and 20% higher in 2025 and 2030, respectively, than in 2020. Emissions of PM, SO_2_, NO_x_, and CO_2_ will be reduced by 71.4%, 77.7%, 51.4%, and 6.7%, respectively, between 2020 and 2030. More effective pollution technologies must be developed. The two emission reduction scenarios predict similar reductions in NO_x_, PM, and SO_2_ emissions, primarily due to the assumed control technologies being near saturation. Advancements in NO_x_ control technologies are promising and should be the primary focus of future research. Improving control technologies will yield better emission reduction outcomes than will changing product structures. Technologies for controlling CO_2_ emissions have not been widely adopted or have not been developed for large-scale applications. The forecast part of this study only considers the emission reduction of various air pollutants in the production process. Emission reductions achieved by adjusting the energy structure will be more effective than those achieved by other methods. The projected emission reductions for each scenario in 2025 and 2030 are displayed in Figure 8.

## 4. Conclusions and Policy Implications

An evaluation of air pollutants and CO_2_ emissions from China’s refractory industry was conducted for the period 2009–2021. The results demonstrated that despite comparable product yield across years, all pollutant emissions significantly decreased over time. PM, SO_2_, and NO_x_ emissions decreased by 7.1% (1515 t), 23.6% (2982 t), and 27.8% (3178 t), respectively. This notable improvement was driven by government policies, advancements in treatment technologies, and the structural adjustments in refractories product. Emissions levels were found to depend on furnace type. Emissions from electric furnaces were only slightly less than emissions from fuel furnaces operating at 1200–1700 °C. Therefore, the use of electric furnaces does not justify the relaxation of regulations or purification measures. The analysis of pollutant-driving factors indicated that the degree of control technology has a major effect on emission reductions. Future efforts should focus on developing cleaner and more effective pollution control technologies, increasing usage of the most efficient equipment, and optimizing terminal treatment facilities. Under the new development strategy of carbon peak and carbon neutrality, China’s steel industry will undergo a massive technological shift and enter a new developmental stage. The refractory manufacturing industry should adapt to the strategic transformation of the steel industry to achieve goals related to a green energy transition. In the next 10 years, long-lasting, functional, and environmentally friendly refractory materials will become the focus of the industry.

## Figures and Tables

**Table 1:** Results of uncertainty analysis of emission inventories.

Items	Calculated Values in the List (t)	Average (t)	95% Confidence Interval (t)	Uncertainty
PM	19,819.46	17,763.30	8976.60–27,472.72	−49.5–54.7%
SO_2_	9642.09	9635.61	5642.75–13,947.66	−41.4–44.8%
NO_x_	8234.61	9276.64	5858.37–12,992.63	−36.9–40.1%
CO_2_	13.07 × 10^6^	13.29 × 10^6^	8.22 × 10^6^–18.76 × 10^6^	−38.2–41.2%

## Data Availability

Data will be made available upon request.

## Supplementary Materials

The following supporting information can be downloaded at: https://www.mdpi.com/article/10.3390/toxics13070533/s1, Figure S1: The production of refractory materials in China from 2009 to 2021; Figure S2: Frequency distribution of emissions simulated by Monte Carlo; Table S1: Production coefficient of air pollutants; Table S2: Removal rate of air pollutants (%); Table S3: CO2 emission factors of various products; Table S4: Output of various refractory materials (10 kt); Table S5: Output of Magnesia-based Refractory Raw Material (10 kt); Table S6: The removal rate of each pollutant under different scenarios (%); Table S7: Forecast of output of various refractory products and raw materials (10 kt); Table S8: Uncertainty parameter Setting [53,54,55,56,57].
