# Supplementary material for: Life Cycle Emissions and Driving Forces of Air Pollutants and CO2 from Refractory Manufacturing Industry in China Based on LMDI Model
Source: Toxics. 2025 Jun 26;13(7):533. doi: 10.3390/toxics13070533 (PMC12300534; doi:10.3390/toxics13070533)
Supplement: Supplementary file 1 [file toxics-13-00533-s001.zip › toxics-3658256-supplementary.pdf]

Supporting Information

Life cycle emissions and driving forces of air pollutants and CO_2 from refractory manufacturing industry in China based on LMDI model

Yan Wang[a, †], Yu Shangguan[b, †], Cheng Wang[c], Xinyue Zhou[b], Huanjia Liu[b], Yi cao[d], Xiayu Liu[b], Yan Guo[b], Guangxuan Yan[b], Panru Kang[e,*], Ke Cheng[b,**]

[a] School of Public health, Xinxiang Medical University, Xinxiang 453003, P.R. China

[b] School of Environment, Key Laboratory of Yellow River and Huai River Water Environment and Pollution Control, Ministry of Education, Henan Key Laboratory for Environmental Pollution Control, Henan Normal University, Xinxiang, Henan, 453007, P.R. China

[c] China National Environmental Monitoring Centre, Beijing, 100012, P.R. China

[d] Institute of Infectious Disease Prevention and Control, Luoyang Center for Disease control and Prevention, Luoyang 471022, P.R. China

[e] State Key Laboratory of Environmental Criteria and Risk Assessment, Chinese Research Academy of Environmental Sciences, Beijing 100012, P.R. China

[†]These authors share first authorship

*Corresponding Author: kangpr@craes.org.cn (Mrs Panru Kang) and chengke@htu.edu.cn (Prof. Ke Cheng)

Content:

Number of pages: 9

Number of Tables: 8

Number of Figures: 2

Table List

Table S1 Production coefficient of air pollutants
Table S2 Removal rate of air pollutants (%)
Table S3 CO_2 emission factors of various products
Table S4 Output of various refractory materials (10 kt)
Table S5 Output of Magnesia-based Refractory Raw Material (10 kt)
Table S6 The removal rate of each pollutant under different scenarios (%)
Table S7 Forecast of output of various refractory products and raw materials (10 kt)
Table S8 Uncertainty parameter Setting

Figure List

Figure S1. The production of refractory materials in China from 2009 to 2021
Figure S2. Frequency distribution of emissions simulated by Monte Carlo

Table S1 Production coefficient of air pollutants

Product	Material	Furnace type	PM (kg/t)	SO_2 (kg/t)	NO_X (kg/t)	Date Source
Alumina Brick, Clay Brick, Silica Brick	Alumina, Clay, Silica + Gas	Tunnel kiln	0.48	3.22	2.08	Pollutant Generation and Discharge of Industrial Product
Alumina Brick, Clay Brick, Silica Brick	Alumina, Clay, Silica + Gas	Intermittent kiln	0.57	4.62	2.92	
Alumina Brick, Clay Brick, Silica Brick	Alumina, Clay, Silica + Coal	Intermittent kiln	4.93	7.24	4.17	
Magnesium Brick	Magnesia-based raw material + Oil	Tunnel furnace	0.76	2.06	1.99	
Carbon Brick	Refractory raw materials + Carbon	Drying kiln	0.42	0.06	0.027	
Dead Burnt Magnesia	Magnesite	Shaft kiln	1.02	0.79	0.93	
Fused Magnesia	Magnesite	Electric arc furnace	2.94	—	—	
Caustic Calcined Magnesia	Magnesite	Light firing kiln	0.42	0.25	0.29	
Other	Refractory raw materials + Gas	Calcining kiln	0.36	2.21	1.88	

Table S2 Removal rate of air pollutants (%)

Material	Furnace type	PM	SO_2	NO_X	Date Source
Alumina, Clay, Silica + Gas	Tunnel kiln	90 (WET)	80 (WFGD)	50 (SNCR)	Pollutant Generation and Discharge of Industrial Product
Alumina, Clay, Silica + Gas	Intermittent kiln	85 (WET)	85 (FD)	50 (SNCR)	
Magnesia-based raw material + Oil	Tunnel kiln	99 (FFs)	85 (FD)	50 (SNCR)	
Magnesite	Furnaces for refractory materials	85 (WET)	60 (FD)	50 (SNCR)	
Refractory raw materials + Gas	Furnaces for refractory materials	99 (FFs)	85 (FD)	50 (SNCR)	

Note: WET denotes wet scrubbers; FFs denotes fabric filters; WFGD denotes wet flue gas de-sulfurization; FD denotes furnace de-sulfurization; SNCR denotes selective non-catalytic reduction.

Table S3 CO_2 emission factors of various products

Product	Material	CO_2(t/t)	Date Source
Magnesium Brick	Magnesia-based raw material + Oil	0.47	Refractory Industry Standards Conditions、Energy Statistics Knowledge Handbook
Alumina Brick	Alumina + Gas	0.52	
Alumina Brick	Alumina + Coal	0.85	
Clay Brick	Clay + Gas	0.28	
Clay Brick	Clay + Coal	0.46	
Silica Brick	Silica + Gas	0.55	
Silica Brick	Silica + Coal	0.90	
Carbon Brick	Refractory raw materials + Carbon	0.08	
Dead Burnt Magnesia	Magnesite	0.52	
Fused Magnesia	Magnesite	0.68	
Caustic Calcined Magnesia	Magnesite	0.52	
Unshaped Refractory	——	0.03	

Table S4 Output of various refractory materials (10 kt)

Year	Clay Brick	Alumina Brick	Silica Brick	Magnesium Brick	Carbon Brick	Unshaped Refractory	Other	Date Source
2009	390.52	209.17	237.61	159.40	245.10	841.21	220.27	China Refractory Industry Yearbooks (2010–2014)、(2015-2018) and current data from the Association of China Refractories Industry
2010	401.93	281.81	247.37	170.23	300.51	1044.74	266.65	
2011	421.96	287.99	263.40	206.54	298.30	1117.13	291.52	
2012	401.20	271.87	241.35	174.49	271.20	1127.78	270.77	
2013	384.41	274.58	232.42	180.35	295.03	1141.81	355.49	
2014	348.18	260.79	211.82	193.71	290.10	1099.35	339.55	
2015	320.41	233.60	187.12	184.83	270.01	1039.98	321.32	
2016	284.32	202.99	116.79	173.83	260.31	985.99	307.90	
2017	274.63	200.85	100.46	164.40	267.40	943.90	283.90	
2018	245.36	186.09	140.61	213.64	276.64	963.74	257.08	
2019	237.59	191.88	145.81	220.75	286.89	1030.49	255.28	
2020	228.30	202.34	156.73	227.98	299.07	1044.85	256.25	
2021	203.29	197.21	162.32	216.22	295.62	1021.18	249.09	

Table S5 Output of Magnesia-based Refractory Raw Material (10 kt)

Year	Dead Burnt Magnesia	Fused Magnesia	Caustic Calcined Magnesia	Date Source
2009	395.74	136.80	342.40	China Refractory Industry Yearbooks (2010–2014)、(2015-2018) and current data from the Association of China Refractories Industry.
2010	438.10	166.30	371.20	
2011	483.58	166.69	402.06	
2012	401.58	145.70	345.96	
2013	424.30	150.90	441.90	
2014	2018.90	672.97	1934.78	
2015	2507.38	835.79	2402.90	
2016	2681.11	893.70	2569.40	
2017	2706.67	902.22	2593.89	
2018	2740.34	913.45	2626.16	
2019	2638.44	879.48	2528.51	
2020	2638.44	879.48	2528.51	
2021	2495.95	831.98	2391.95	

Table S6 The removal rate of each pollutant under different scenarios (%)

Year	Scenario	PM	NOx	SO_2
2025	ERS	99.1	75.8	89.1
	SRS	99.4	83.3	94.6
2030	ERS	99.2	78.3	92.5
	SRS	99.5	86.7	95.0

Table S7 Forecast of output of various refractory products and raw materials (10 kt)

Year	Clay Brick	Alumina Brick	Silica Brick	Magnesium Brick	Unshaped Refractory	Other	Dead Burnt Magnesia	Fused Magnesia	Caustic Calcined Magnesia
2025	162.29	166.31	117.46	240.84	1006.74	240.48	557.36	182.36	578.00
2030	115.37	136.70	88.03	254.42	970.01	227.10	586.35	188.30	658.00

Table S8 Uncertainty parameter Setting

Key parameters		Data distribution	Sources
Activity level	Clay Brick	Normal (CV: 15%)	[1-5]
	Alumina Brick	Normal (CV: 15%)	
	Silica Brick	Normal (CV: 15%)	
	Magnesium Brick	Uniform	
	Carbon Brick	Triangular	
	Unshaped Refractory	Normal (CV: 15%)	
	Other	Uniform	
Emission factors	PM	Normal (CV: 10%)	
	SO_2	Normal (CV: 10%)	
	NO_X	Normal (CV: 10%)	
Pollutant removal efficiency	ESP	Normal (CV: 10%)	
	FFs	Normal (CV: 10%)	
	EFs	Normal (CV: 10%)	
	WET	Triangular	
	WFGD	Normal (CV: 10%)	
	FD	Normal (CV: 10%)	

LNB	Triangular
SCR	Normal (CV: 10%)
SNCR	Normal (CV: 10%)

Note: ESP denotes electrostatic precipitator; FFs denotes fabric filters; EFs denotes electrostatic fabric filters; WET denotes wet scrubbers; WFGD denotes wet flue gas de-sulfurization; FD denotes furnace de-sulfurization; LNB denotes low NOx burner; SCR denotes selective catalytic reduction; SNCR denotes selective non-catalytic reduction.

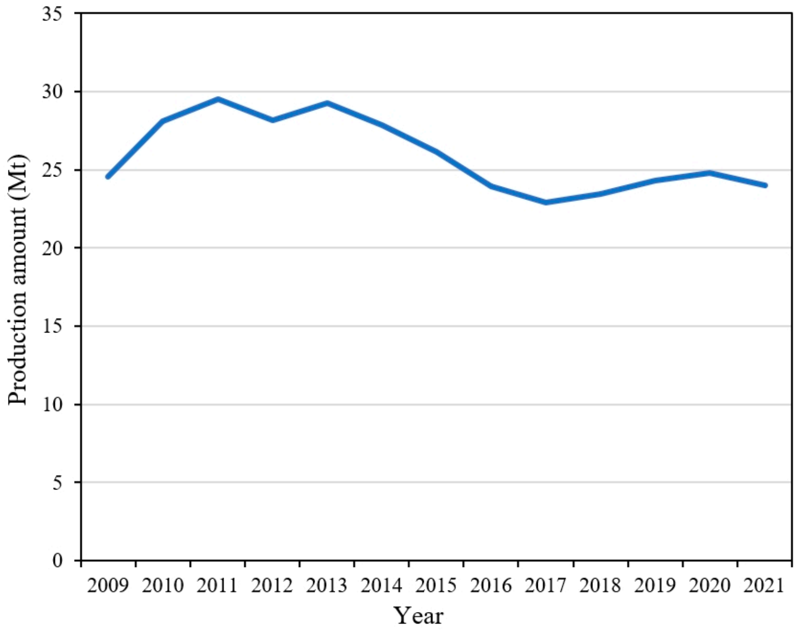

Figure S1. The production of refractory materials in China from 2009 to 2021

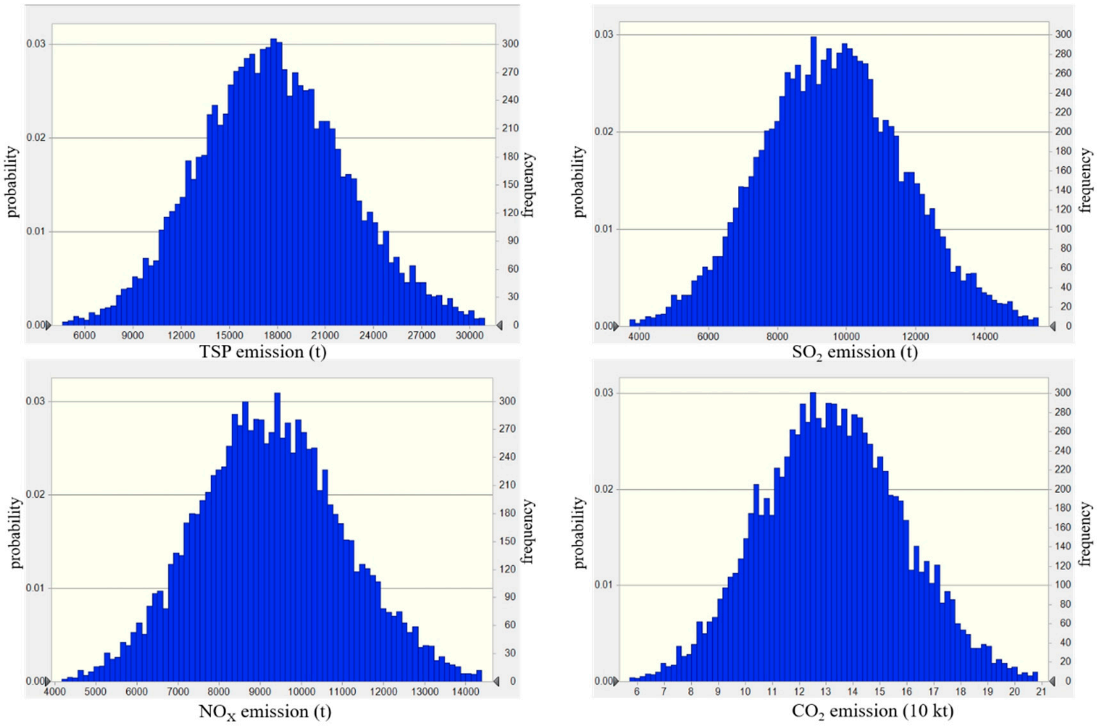

Figure S2. Frequency distribution of emissions simulated by Monte Carlo

References

52. Wang, Y.; Cheng, K.; Wu, W.; Tian, H.; Yi, P.; Zhi, G.; Fan, J.; Liu, S. Atmospheric emissions of typical toxic heavy metals from open burning of municipal solid waste in China. Atmos. Environ. 2017, 152, 6-15, doi:https://doi.org/10.1016/j.atmosenv.2016.12.017.
53. Xue, Y.; Nie, L.; Zhou, Z.; Tian, H.; Yan, J.; Wu, X.; Cheng, L. Historical and future emission of hazardous air pollutants (HAPs) from gas-fired combustion in Beijing, China. Environ. Sci. Pollut. Res. 2017, 24, 16946-16957, doi:https://doi.org/10.1007/s11356-017-9260-6.
54. Gao, J.; Wang, K.; Tong, Y.; Yue, T.; Wang, C.; Zuo, P.; Liu, J. Refined spatio-temporal emission assessment of Hg, As, Cd, Cr and Pb from Chinese coal-fired industrial boilers. Sci. Total Environ. 2021, 757, 143733, doi:https://doi.org/10.1016/j.scitotenv.2020.143733.
55. Tong, Y.; Gao, J.; Wang, K.; Jing, H.; Wang, C.; Zhang, X.; Liu, J.; Yue, T.; Wang, X.; Xing, Y. Highly-resolved spatial-temporal variations of air pollutants from Chinese industrial boilers. Environ. Pollut. 2021, 289, 117931, doi:https://doi.org/10.1016/j.envpol.2021.117931.
56. Wang, K.; Tong, Y.; Yue, T.; Gao, J.; Wang, C.; Zuo, P.; Liu, J. Measure-specific environmental benefits of air pollution control for coal-fired industrial boilers in China from 2015 to 2017. Environ. Pollut. 2021, 273, 116470, doi:https://doi.org/10.1016/j.envpol.2021.116470.
